# Use of food frequency questionnaire to assess relationships between dietary habits and cardiovascular risk factors in NESCAV study: validation with biomarkers

**DOI:** 10.1186/1475-2891-12-143

**Published:** 2013-11-06

**Authors:** Nicolas Sauvageot, Ala’a Alkerwi, Adelin Albert, Michèle Guillaume

**Affiliations:** 1 CRP-Santé, Department of Public Health, 1A rue Thomas Edison, L-1445 Strassen, Luxembourg; 2 Ecole de Santé Publique, Université de Liège, Liège, Belgium; 3 CRP-Santé, CCMS (Competence center in methodology and statistics), 1A rue Thomas Edison, L-1445 Strassen, Luxembourg

**Keywords:** Food frequency questionnaire, Validation, Cardiovascular risk, Biomarkers, NESCAV

## Abstract

**Background:**

Validation of Food frequency questionnaire (FFQ) is particularly important element, as incorrect information may lead to false associations between dietary factors and diseases. The aim of the study was to evaluate the validity of the FFQ used in NESCAV (Nutrition, Environment and Cardiovascular Health) study, by comparing the estimated intakes of fruits and vegetables and of several micro-nutrients with corresponding nutritional biomarkers.

**Methods:**

Relative validity was assessed in a sample of 922 subjects (452 men and 470 women). Comparisons between FFQ-estimates and their corresponding biomarkers were performed through correlation and cross classification into quintiles by using both crude and energy-adjusted FFQ-estimates. Correlations adjusted for confounders were also computed. All analyses were performed separately for men and women.

**Results:**

Concerning micro-nutrients, significant correlations were found for vitamin B9, D, E, B12 β-carotene and iodine in both men and women. Energy-adjustment led to an increase of all correlations cited previously. However, after excluding supplement users, correlations for vitamin D were not significant anymore. Concerning fruits and vegetables, all correlations were significant. Vegetables alone and fruits and vegetables correlated better in men (r around 0.2) than in women (r around 0.1). In men, correlation was also better for vegetables alone and fruits and vegetables than fruits alone.

**Conclusion:**

These data demonstrate that this FFQ is a reasonable tool to assess intakes of fruits and vegetables and of several micro-nutrients. We conclude that our FFQ is suitable to be used in NESCAV study, although protein and vitamin D estimates should be interpreted with caution.

## Background

The measurement of dietary intakes is considered as the greatest challenge to nutritional epidemiology [1]. The food frequency questionnaire (FFQ) is one of the most common tools used in large-scale population-based studies to examine the relation between diet and disease, owing to easy administration and low cost [2,3]. But like all dietary methods, estimates derived from FFQ data suffer from random and systematic error and may not represent adequately the habitual food intake [4]. Therefore, a prior validation of FFQ is particularly important element, as incorrect information may lead to false associations between dietary factors and diseases [5].

In epidemiological studies, the odd ratio or relative risk are the most common measures of association between disease and nutrient intake. Accordingly, FFQs must be able to rank individuals along the distribution of intake, so that individuals with low intakes can be separated from those with high intakes [6,7].

The relative validity of the FFQs is usually assessed by comparing their data with those of food records, because a gold standard as a “truth reference” is not available. However, food records may introduce a bias as they also suffer from measurement errors that are likely to be correlated with errors in FFQ [8]. Because the recording of food intake is subject to behavioral modification, subjects may reduce their dietary intake while keeping food records [9]. Moreover, nutrient intakes estimates are also based on the same food composition data. Since both self-reported methods contain similar errors, resulting in flawed estimates of validity, biomarkers of dietary intake have become increasingly important in nutritional validation studies [10]. They provide an objective measure of intake of which the errors are largely independent of the errors associated with FFQ [5,11].

In an interregional cross-sectional study NESCAV (Nutrition, Environment and Cardiovascular Health), we used a modified semi-quantitative FFQ to assess dietary habits of the Greater region’s population (Luxembourg, Wallonia in Belgium and Lorraine in France) and to explore the relationship between diet and cardiovascular risk factors (CVRF) [12]. This FFQ was first developed by a Canadian group to assess dietary habits among Quebecois, and was previously validated in this population [13]. In order to achieve the objectives of the NESCAV study, this FFQ was modified and the list of food items was extended. Therefore, new validation studies are necessary [14]. For this purpose, 2 approaches have been applied to investigate the relative validity of the modified FFQ: first, by comparing FFQ data with data from 3-day dietary records (DR), and second, by comparing FFQ data against nutritional biomarkers.

The validation of our modified FFQ against DR has been examined in a previous study [15], where reasonable estimates of validity have been shown for most nutrients, although probably overestimated.

In the present study, we further evaluated the validity of consumption of fruits and vegetables and of several micronutrients in comparison with measures of corresponding nutritional biomarkers.

## Methods

### NESCAV study

In the frame of European INTERREG IV A program, 2007–2013, a cross-border project entitled “Nutrition, Environment and Cardiovascular Health NESCAV” has been initiated to monitor the cardiovascular health and risk factors profile of the Greater region’s population, by using standard methodology and instruments for data collection. A more detailed description of the original study has been published elsewhere [12]. The study was approved by the institutional ethics committees and all participants provided written informed consent.

### Validation sample selection

A total of 984 subjects, aged 18–69 years, recruited from the Wallonia region to participate to NESCAV study were used in this analysis.

### Nutritional assessment

#### ***Food frequency questionnaire***

In NESCAV study, the dietary habits were assessed by using a modified semi-quantitative FFQ. The concept and rationale for major food groups has been developed, basing on the validated Canadian FFQ, which was composed of 73-food items to capture food consumption among adults living in Quebec [13]. Our FFQ has been adapted to the studied population’s cultural and linguistic particularities, to assess the subjects’ intake of energy and nutrients coming from different cultural backgrounds. For this purpose, the list of food-items was extended by integrating new foods to cover the diversity of dietary habits of the Greater Region’s population.

The developed version of the FFQ aims to assess the dietary intake, by asking the participants to report the frequency of consumption and portion size of approximately 134-line items over the last three months. Each item is defined by a series of foods or beverages which are categorized into 9 major food groups: starchy food, fruits, cooked and raw vegetables, meat-poultry-fish-eggs, prepared dishes, dairy products, fats, drinks (alcoholic and non-alcoholic), and miscellaneous. The Participants reported the frequency of consumption of each food group on the basis of 6 levels of frequencies: rarely or never; one to three times a month; one to two times a week; three to five times a week; one time a day; 2 times or more a day. Standard serving sizes and food models based on a photographic manual, validated by the 'SUpplementation en VItamines et Mineraux AntioXydants’ SU.VI.MAX study [16], are provided as a reference to aid the participants to estimate the portion size.

The frequency of consumption of food items was multiplied by the portion size to calculate the grams of food consumed per day. The values used for each frequency option were the following: Never = 0; one to three times a month = 2/30; one to two times a week = 1.5/7; three to five times a week = 4/7; one time a day = 1; 2 times or more a day =2.5. The food items (g/d) were subsequently converted into daily nutrients intake by using the French (SU.VI.MAX) Food Composition Database [17]. Daily intakes of nutrient for individual FFQ items were then summed to obtain daily intake of each nutrients.

The accessibility and readability of our FFQ were assessed in a pre-test phase, on a sample of multicultural group. Given the multi-linguistic nature of the population residing in Luxembourg, the FFQ was translated from French into the three other most used languages, namely German, English and Portuguese, and then backward translated into French to ensure the linguistic validity [18].

#### ***Supplements intake***

Detailed information on micro-nutrients supplement use was also collected, including product name, type of medication, dose, frequency and duration of use. To calculate the daily intake from supplements for a specific micro-nutrient, the daily frequency of use was multiplied by the number of tablet taken per day and by its nutrient composition. Total micro-nutrient intake was then calculated for each micro-nutrient as the sum of dietary and supplemental intake. We designated subjects as supplement users for a specific micro-nutrient if, they declared taking supplement containing this specific micro-nutrient.

#### ***Nutritional biomarkers***

A venous blood sample was drawn from the arm of each subject in sitting position by antecubital vein puncture, after an overnight 8-hour fast. The blood samples were transferred to the core laboratory in tank containing ice packs to maintain a suitable temperature. They were centrifuged within maximum 4 hours after extraction and then immediately analyzed. A morning spot-urine was also drawn. The Laboratory applies strict internal and external standard quality control techniques and performed the measurements of the following biomarkers: vitamin B12 (pg/ml), serum folates (ng/ml), erythrocyte folates (ng/ml), 25-OH vitamin D (ng/ml), α-tocopherol (mg/L), iron (μmol/L), ferritin (ng/ml), transferrin (g/L), iodine/creatinine ratio (μg/g creat), β-carotene (mg/L) and sodium in urine (mmol/L). Further detailed information about specimen collection and laboratory analyses are presented in Additional file 1: Table S1. Table 1 lists the nutrients measured in this study with their corresponding biomarkers.

**Table 1:** FFQ estimates and their corresponding biomarkers

	**FFQ estimates**	**Biomarkers of nutrient status**
Food groups	Fruits (g/day)	β-carotene (mg/L)
	Vegetables (g/day)	β-carotene (mg/L)
	Fruits and vegetables (g/day)	β-carotene (mg/L)
Micro-nutrients	Vitamin B12 (μg)	B12 (pg/ml)
	Vitamin B9 (μg)	Serum folates (ng/ml)
	Vitamin B9 (μg)	Erythrocyte folates (ng/ml)
	Vitamin D (μg)	25-OH Vitamine D (ng/ml)
	Vitamin E (mg)	α-tocopherol (mg/L)
	Iron (mg)	Iron (μmol/L)
	Iron (mg)	Ferritin (ng/ml)
	Iron (mg)	Transferrin (g/L)
	Iodine (μg)	Iode/Creatinine ratio (μg/g)
	β-carotene (μg)	β-carotene (mg/L)
	Sodium (mg)	Urine sodium (mmol/L)

### Statistical analysis

All analyses were performed separately for men and women.

#### ***Exclusion criteria***

Participants who reported incomplete information on the frequency of use and/or the product name of multivitamin supplements were excluded.

#### Descriptive analysis

As most data were skewed, median, 25^th^ and 75^th^ percentiles were presented. Socio-demographic characteristics, FFQ derived estimates and nutritional biomarkers were compared between men and women by using Kruskal-Wallis and Chi-squared tests for continuous and categorical variables, respectively.

Since both absolute individual nutrient intakes and nutrient composition of the diet will be used in NESCAV study, all nutrients were energy-adjusted according to the regression residual method of Willet and Stampfer [19]. In addition, three lipid-soluble biomarkers, namely: plasma α-tocopherol, 25-OH vitamin D and plasma β-carotene were “lipid adjusted” by using residuals method calculated by regressing biomarkers on the sum of plasma cholesterol and triglycerides [20-24].

#### ***Correlation***

Three models of analyses were built, namely crude, energy adjusted model I and energy-adjusted model II for both men and women. Firstly, in the crude model, comparisons of crude FFQ intakes with corresponding biomarkers were assessed by Spearman correlation coefficients. Secondly, in the energy-adjusted model I, correlations between energy-adjusted FFQ intakes and biomarkers were computed. Finally, in order to explain the potential variation in blood levels of the nutrients, correlations between energy-adjusted intakes and their corresponding biomarkers were adjusted for several confounders (energy-adjusted model II), which were determined according to the literature [22,25]. Those potential confounders were age, body mass index (BMI), current smoking and specific micro-nutrient supplement use.

#### ***Cross-classification into quintiles***

For each association, the distributions of FFQ and biomarkers results were divided into quintiles. Individual results were then cross-classified in the FFQ and biomarkers categories, and the percentage of subjects classified in the same quintile, within one quintile (in the same/adjacent) were considered as measure of its capacity of ranking. The proportion of FFQ subjects falling in opposite categories was also computed yielding an estimation of grossly misclassification errors. Moreover, the agreement between the 5 categorical scales was measured by the weighted Cohen kappa coefficient (ĸ); the weighting factors being 1 for complete agreement (same category), 0.5 for disagreement one category apart (adjacent categories) and 0 for complete disagreement (opposite categories).

#### ***Identification of factors associated with the validity of the FFQ***

To identify factors associated with the validity of FFQ intake estimates, a logistic regression model was performed with the agreement within one quintile as the dependent variable and personal characteristics of participants as explanatory variables. These included age (as continuous variable), obese status (obese, non-obese), current smoking (smoker, nonsmoker), educational level (University/ Not University level), and specific micro-nutrient supplement use (use of the specific micro-nutrient supplement/no use).

#### ***Sensitivity analyses***

In order to evaluate if supplement users affect significantly the results on micro-nutrients, all analysis described above were performed again excluding supplement users.

The same sub analysis was also performed to assess if misreporters of total energy intake affect results. We identified plausible misreporters of total energy intake using the method of Golberg namely the ratio of reported energy intake to estimated energy requirement (EER) [26]. EER was estimated using published equations based on gender, age, weight, height and physical activity level (PAL). A constant value was assumed for PAL. A 95% confidence interval was created around the log of the ratio, and individuals who felt outside of the confidence interval were classified as under or over reporters.

According to the work of Krall et al. [27], we examined the relationship between vitamin D intake and 25-OH vitamin D concentrations in the subset of individuals who were seen during late winter and early spring (February to May), in order to adjust for the effect of sunlight exposure on plasma vitamin D concentrations. Moreover, individuals who use solarium were excluded from this analysis.

All analyses were performed using SAS statistical software (version 9.2, SAS Institute Inc).

## Results

### Data cleaning

The initial data-base was constituted of 984 individuals. Because of lack of information on micro-nutrients use, 62 participants respectively were excluded. Therefore 922 participants (452 men and 470 women) were available for the analysis.

### ***Descriptive analysis***

Table 2 describes the characteristics of the sampled subjects, nutrient intake from the FFQ and the nutritional biomarkers concentrations. The distribution between men and women was comparable in terms of age with around 18% of 18–29 y, 43% of 30–49 y and 39% of 50–69 y. Women were significantly more educated than men with 49.1% of women having a superior diploma against 41.9% of men (P = 0.028). The proportion of obese and smokers were similar between men and women. There were 17.3% of obese and 23.01% of smokers among women against 20.04% and 27.66%, respectively, among men.

**Table 2:** Socio-demographic characteristics, FFQ-derived estimates and nutritional biomarkers of participants enrolled in the validation study by sex

**Characteristics**	**Women (n =452)**			**Men (n =470)**			
	**No.**	**%**	**Median [P**_**25**_**; P**_**75**_**]**	**No.**	**%**	**Median [P**_**25**_**; P**_**75**_**]**	**P-value**
Age. years							NS
18-29	82	18.1		78	16.6		
30-49	193	42.7		207	44.04		
50-69	177	39.2		185	39.4		
Education							0.028
No superior diploma	230	50.9		273	58.1		
Superior diploma	222	49.1		197	41.9		
Obese^a^	78	17.3		94	20.04		NS
Smokers	104	23.01		130	27.66		NS
Use of supplement							
Vitamin B12	3	0.66		7	1.49		NS
Vitamin B9	9	1.91		8	1.77		NS
Vitamin E	10	2.21		9	1.91		NS
Vitamin D	77	17.04		34	7.23		<0.0001
Iron	8	1.77		7	1.49		NS
Iodine	5	1.11		7	1.49		NS
β-carotene	5	1.11		2	0.43		NS
FFQ-estimated daily dietary intake							
Food groups							
Fruits (g/day)			145.2 [49.3; 299.2 ]			91.2 [19.6; 252.2]	0.0002
Vegetables (g/day)			51.8 [25.1; 103.4 ]			35.9 [18.4; 72.1]	<0.0001
Fruits and vegetables (g/day)			211.8 [108.7; 383.2 ]			163.8 [67.07; 308.9 ]	<0.0001
Micro-nutrients							
Vitamin B12 (μg)			4.7 [3.4; 6.3]			6 [4.5; 8.1]	<0.0001
Vitamin B9 (μg)			315.8 [246.6; 407.2]			358 [286.2; 451.4]	<0.0001
Vitamin E (mg)			12.6 [10; 16.6]			14.1 [11; 18.9]	0.0006
Vitamin D (μg)			2.8 [1.4; 6]			2.8 [1.6; 4.8]	NS
Iron (mg)			13.4 [10.7; 16.2]			16.1 [13.5; 19.8]	<0.0001
Iodine (μg)			127 [100.3; 170.7]			146.3 [119.2; 197.6]	<0.0001
β-carotene (μg)			3818.6 [2597.6; 5464.4]			3655.4 [2334.6; 5392]	0.04
Sodium (mg)			3399.1 [2707.4; 4090.7]			4326.68 [3418.8; 5343]	<0.0001
Blood levels of biomarkers							
B12 (pg/ml)			391.8 [302.1; 530]			409.75 [326.8; 514.9]	NS
Serum folates (ng/ml)			7.8 [6.2; 10.2]			7.16 [5.6; 9.7]	0.015
Erythrocyte folates (ng/ml)			586.7 [509.6; 676.7]			589 [503; 705.4]	NS
25-OH Vitamine D (ng/ml)			22.1 [15.4; 29.1]			17.6 [11.9; 25]	<0.0001
α-tocopherol (mg/L)			12.8 [10.8; 15.4]			12.4 [10.4; 14.9]	NS
Iron (μmol/L)			17.6 [13.7; 22]			19.1 [15.3; 23.9]	0.0004
Ferritin (ng/ml)			69.9 [37.7; 129.2]			199.1 [121.2; 326.5]	<0.0001
Transferrin (g/L)			2.8 [2.5; 3.1]			2.6 [2.4; 2.9]	<0.0001
Iode/Creatinine ratio (μg/g creat)			70 [51; 105]			72 [53; 97]	NS
Urine sodium (mmol/L)			113 [81; 154]			134 [100; 166.1]	<0.0001
β-carotene (mg/L)			0.23 [0.12; 0.37]			0.15 [0.08; 0.24]	<0.0001

^a^Obesity was defined as a body mass index (weight(kg)/height(m)^2^) ≥ 30.

NS: Not significant.

With regard to supplements use, 17.04% of women reported using vitamin D supplement against only 7.23% of men. For the use of others micro-nutrient supplements, the proportions were all inferior to 2% in both men and women. Compared to men, women reported significantly higher intakes of fruits and vegetables. For the micro-nutrients, men reported higher intakes of vitamin B12, B9, E, iron, iodine and β-carotene and sodium. Concerning biomarkers, blood level of serum folates, 25-OH vitamin D, transferrin and β-carotene were higher in women while iron, ferritin and urine sodium were superior in men (Table 2).

### Correlation

Correlations between biomarker measurements and crude and energy-adjusted FFQ estimates, for both men and women, were presented in Table 3. In the crude model, significant correlations were found for fruits alone, vegetables alone, fruits and vegetables, vitamin B9, D, iodine and β-carotene in both men and women. Vitamin B12, E and sodium were correlated with their biomarkers only in women.

**Table 3:** Correlations between FFQ-derived estimates and biomarkers

			**Men**			**Women**		
**FFQ-estimated daily dietary intakes**		**Biomarkers**	**Crude**	**Energy-adjusted model I**	**Energy-adjusted model II**^**£**^	**Crude**	**Energy-adjusted model I**	**Energy-adjusted model II**^**£**^
Food groups	Fruits (g)	β-carotene (mg/L)	0.12†	0.13†	0.11†	0.15†	0.14†	0.1†
	Vegetables (g)	β-carotene (mg/L)	0.17‡	0.18‡	0.18‡	0.11†	0.08†	0.11†
	Fruits and vegetables (g)	β-carotene (mg/L)	0.18‡	0.19‡	0.17‡	0.14†	0.13†	0.1†
Micro-nutrients	Vitamin B12 (μg)	B12 (pg/ml)	0.06	0.13†	0.12†	0.11†	0.1†	0.09†
	Vitamin B9 (μg)	Serum folates (ng/ml)	0.27‡	0.35‡	0.29‡	0.28‡	0.36‡	0.32‡
	Vitamin B9 (μg)	Erythrocyte folates (ng/ml)	0.28‡	0.32‡	0.25‡	0.23‡	0.36‡	0.25‡
	Vitamin D (μg)	25-OH Vitamine D (ng/ml)	0.26†	0.28†	0.07†	0.55‡	0.54‡	0.06
	Vitamin E (mg)	α-tocopherol (mg/L)	0.05	0.13†	0.09†	0.11†	0.11†	0.05
	Iron (mg)	Iron (μmol/L)	-0.08	-0.04	-0.05	0.09†	0.07	0.06
	Iron (mg)	Ferritine (ng/ml)	-0.04	0.04	0.02	0.004	0.007	0.03
	Iron (mg)	Transferrine (g/L)	0.04	0.03	0.01	0.01	0.03	0.02
	Iodine (μg)	Iode/Creatinine ratio (μg/g)	0.11†	0.15†	0.11†	0.2‡	0.23‡	0.22‡
	β-carotene (μg)	β-carotene (mg/L)	0.18‡	0.2‡	0.2‡	0.22‡	0.22‡	0.22‡
	Sodium (mg)	Urine sodium (mmol/L)	0.06	0.1†	0.07	0.11†	0.12†	0.1

^£^Correlations were adjusted for age, BMI, current smoking and specific micro-nutrient supplement use.

‡P < 0.001.

†P < 0.05.

Concerning energy adjusted model I, overall, we noticed an increase of all previously cited correlations in both men and women. Except for vitamin D, E and sodium adjustment for confounders did not change the correlations coefficients (energy adjusted model 2). Indeed, after adjustment for confounders, vitamin D intake was no longer correlated with blood levels and correlation coefficients decreased from 0.28 to 0.07 in men and from 0.54 to 0.06 in women. For vitamin E and sodium, correlations were also significantly decreased. In all models, better correlations were observed for vitamin B9 intake (r around 0.3). For β-carotene, all correlations were around 0.20 in both men and women. Compared to the others micro-nutrients, iodine, vitamin B12 and E intakes were also significantly correlated to biomarkers, but their correlations were lower (r around 0.10). However, iodine intake correlated better in women (r around 0.2). Concerning iron intake, no significant associations were found with the three biomarkers used.

For fruits and vegetables, all correlations were significant. Vegetables alone and fruits and vegetables correlated better in men (r around 0.2) than in women (r around 0.1). In men, correlation was also better for vegetables alone and fruits and vegetables than fruits alone.

### Cross-classification into quintiles

Table 4 presents the results of the cross-classification into quintiles between biomarkers measurement and FFQ-derived estimates, for both men and women. As seen with correlations coefficients (Table 3), vitamin B9 was the FFQ-derived estimates which agreed the best with its biomarkers. Percentages of subjects within the same quintile were the highest (> 60%) and percentages of extreme classification were the lowest. Kappa coefficients were also the highest (For serum folates, ĸ = 0.21 in men and 0.23 in women; for erythrocyte folates, ĸ = 0.19 in men and 0.21 in women). As in correlation analysis, FFQ-derived iodine and β-carotene estimates agreed well with corresponding biomarkers (ĸ above 0.10). Vitamin B_12_, Vitamin E and sodium showed ĸ between 0.05 and 0.10 whereas all ĸ concerning iron were inferior to 0.05.

**Table 4:** Cross-classification into quintiles between FFQ-derived estimates and biomarkers (Energy-adjusted data)

			**Men**				**Women**			
**FFQ-estimated daily dietary intakes**		**Biomarkers**	**Same (%)**	**Within one (%)**	**Extreme (%)**	**ĸ**	**Same (%)**	**Within one (%)**	**Extreme (%)**	**ĸ**
Food groups	Fruits (g/day)	β-carotene (mg/L)	20.96	54.9	5.01	0.07	24.28	56.25	5.05	0.1
	Vegetables (g/day)	β-carotene (mg/L)	23.23	59.22	5.47	0.11	25.72	55.77	6.91	0.07
	Fruits and vegetables (g/day)	β-carotene (mg/L)	21.64	57.63	4.56	0.1	23.32	54.81	5.05	0.08
Micro-nutrients	Vitamin B12 (μg)	B12 (pg/ml)	23.56	54.04	6.24	0.07	23.54	55.34	6.31	0.09
	Vitamin B9 (μg)	Serum folates (ng/ml)	25.35	64.52	3.23	0.21	27.1	65.95	2.64	0.23
	Vitamin B9 (μg)	Erythrocyte folates (ng/ml)	25.87	62.82	4.85	0.19	29.88	61.45	2.65	0.21
	Vitamin D (μg)	25-OH Vitamine D (ng/ml)	29.2	68.14	7.08	0.19	37.7	67.21	0	0.37
	Vitamin E (mg)	α-tocopherol (mg/L)	25.29	60.69	6.44	0.11	21.65	54.98	6.33	0.06
	Iron (mg)	Iron (μmol/L)	19.59	51.03	9.11	-0.02	18.55	54.21	6.99	0.03
	Iron (mg)	Ferritin (ng/ml)	23.69	55.13	8.2	0.05	19.95	53.12	7.93	0
	Iron (mg)	Transferrin (g/L)	20.96	53.99	7.97	0.03	21.45	53.98	7.23	0.03
	Iodine (μg)	Iode/Creatinine ratio (μg/g)	21.5	58.42	4.21	0.1	24.56	59.5	4.81	0.14
	Sodium (mg)	Urine sodium (mmol/L)	22.81	53.51	6.58	0.06	19.08	54.02	5.52	0.05
	β-carotene (μg)	β-carotene (mg/L)	24.36	56.84	4.87	0.13	22.79	59.55	4.41	0.13

K: Kappa coefficient.

Concerning fruits and vegetables intakes, as seen with correlations coefficients (Table 3), vegetables alone and fruits and vegetables intakes agreed better with β-carotene in men. Agreement was also better for vegetables alone and fruits and vegetables than fruits alone in men.

### Identification of factors associated with the validity of the FFQ

Concerning factors associated with the agreement between FFQ estimates and biomarkers, we found that for vitamin E intake and α-tocopherol, smokers were more likely to be correctly classified in the same/adjacent quintile, likewise, for folate and vitamin D supplements’ users (Data not shown).

### Sensitivity analyses

After excluding individuals using specific supplement, the adjusted correlations coefficients were not appreciably changed. The difference between correlations coefficients computed on the total sample and after excluding supplement users were all within 0.02 (data not shown). Concerning the results of cross-classification into quintiles, excluding supplement users did not change significantly the percentages and kappa values; expect for vitamin D. Changes were all within 1% for the percentages and within 0.01 for kappa values (data not shown). However, for vitamin D, ĸ decreased from 0.19 to 0.10 in men and from 0.37 to -0.012 in women, after excluding the supplements users (data not shown). Likewise, for vitamin D, percentages of subjects classified in the same quintile decreased from 29.2% to 19.4% in men and from 37.7% to 22.5% in women.

Likewise, exclusion of the 48 misreporters of energy intake did not significantly change correlations and agreement.

## Discussion

The aim of this study was to evaluate the performance of a modified FFQ used in the NESCAV study, against several nutritional biomarkers. Overall, our FFQ performed well in assessing intakes of fruits and vegetables and several micro-nutrients as correlations were within the range noted by others investigators [2,25,28-35].

Although, biomarkers provide an objective measure of intake of which the errors are largely independent of the errors associated with FFQ [5,11], they have several drawbacks. Indeed, while FFQ measures intake, biomarkers measures circulating concentrations that are influenced not only by dietary intake but also by a number of physiological and environmental factors [36]. The effects of genetic, lifestyle, physiologic and others dietary factors may also bear on the relationship between the amount ingested and the biochemical measurement [11,37]. Although, some of these factors were taken into account by statistical adjustment or restriction, the weak correlations observed between biomarkers and the FFQ-estimated values would be related to their effect [38]. We thus consider the observed correlations to be the lower limit of the ability of the FFQ to measure considered nutrients.

Plasma β-carotene is very sensitive to dietary intake as it is not closely regulated by a homeostatic mechanism [39]. Similar to other studies [31-33], we found correlations around 0.20.

Concerning estimates of fruits and vegetables, although others authors reported higher correlations [40-43], significant positive correlations were found between plasma β-carotene concentration and estimated intakes of vegetables and fruits (r = 0.17 in men and r = 0.1 in women), vegetables alone (r = 0.18 in men and r = 0.11 in women) and fruits alone (r = 0.11 in men and r = 0.1 in women).

For Vitamin B9 intake, we used both serum folate which indicates recent dietary folate intake, and erythrocytes folate, which is an indicator of long-term status [44]. Of the nutrient we considered, folates displayed the highest correlation and kappa coefficients. In men, we obtained adjusted-correlations of 0.25 and 0.29 for energy-adjusted erythrocytes and serum folates respectively. In women, these correlations were equal to 0.25 and 0.32 respectively. These correlations were of the same magnitude to those obtained in previous studies [28] and even higher than others [29,30].

For vitamin B12, the observed correlation, around 0.10 suggests a responsiveness of this biomarker to dietary intake. This result was in the range of other studies excluding supplement users [2].

A single measurement of plasma α-tocopherol, adjusted for blood lipids, appears to be able to represent long-term vitamin E intake to a modest degree [45-47]. However, in several studies, even if total vitamin E intake was positively associated with plasma concentrations of α-tocopherol, this was primarily due to vitamin E supplements since no[38,48,49] association were observed among persons who do not use vitamin E-containing supplements. As most of these studies, our correlations coefficients decreased significantly when adjusted for the use of vitamin E supplement (from 0.13 to 0.09 in men; from 0.11 to 0.05 in women). It is possible that there is a poor relationship between intake and serum levels at the range of intakes from diet alone and a good relationship at the range of intakes achieved through dietary supplements use.

Because plasma vitamin D concentrations are influenced by both diet and sunlight exposure [50], validation of vitamin D intake was undertaken in the late winter when sun synthesis of vitamin D would not be a confounder [27]. In late winter, skin production of vitamin D is nearly null and previous serum vitamin D stores had been depleted or nearly depleted. The observed energy-adjusted correlations (0.28 in men and 0.54 in women), between intake and biomarker suggest a responsiveness of plasma concentrations to dietary intake. But these associations were no longer significant when adjusted for use of vitamin D supplements. Since others [2,51] reported significant correlations, even after excluding supplement users, we think that our FFQ is not performing well in ranking individuals according to vitamin D intake.

Concerning correlations between urinary NA level and sodium intake, we found correlations of 0.10 in women and equal to 0.07 in men. This is not surprising since there are no questions on sodium intake or use of table salt in our FFQ. Similar validation studies assessing the association between FFQ-derived estimates of NA and urinary NA levels are rare. In a Brazilian study among hypertensive subjects [52], the FFQ revealed no significant correlations with 24 h urinary Na while another one [53] found only weak correlation (r = 0.29). An accurate assessment of Na intake implies identification of the sources of Na in the standard diet, yet this is different from the situation for other nutrients which are supplied largely by intrinsic nutrients in specific foods. Na constitutes a part of almost all fresh foods, and it is a major component of industrialized canned and pre-prepared foods. Moreover, table salt and that added when preparing foods are also an important source of Na in the individual diet. Therefore, in order to estimate Na consumption, it is necessary to consider all different sources of dietary Na. It is widely recognized that the observed weak relation between dietary and urinary sodium is attributed to the poor assessment of salt intake by dietary assessment methods, the lack of inclusion of foods prepared with salt in food-composition tables, and the high within-person variability of urinary sodium [54,55]. It is therefore no surprise that the association between FFQ estimate of sodium intake and excretion is so weak.

In our study, no correlation was found between dietary iron intake and the three selected biomarkers: iron, serum ferritin and transferrin. Some investigators have found correlations between dietary iron intake or iron-rich foods and serum ferritin levels [56,57]. Reliable surrogate biomarkers of the total quantity of dietary iron are unavailable because of the wide variation in bioavailability of dietary iron (for instance heme vs non-heme iron), inter-individual variation in biological availability of dietary iron, interactions between dietary iron and absorption enhancers and inhibitors, variations in physiological (menstruation, childbirth) or unphysiological (blood donation) iron losses and uncertain food composition data. Therefore, the lack of correlation for iron and its biomarkers is probably because of the insensitivity of plasma concentrations to intake of iron.

Concerning urine iodine:creatinine ratio, correlations were higher in women (r > 0.20) than in men (r around 0.10). Although, one study reported a correlation coefficient of 0.66 [58], our results are similar to those previously published (r = 0.16 (*34*), r = 0.24(*35*)).

The main strength of this study is that we compared FFQ-estimated intakes with objective biochemical measurements. Although these analyses give the lower limit of the FFQ validity, they allow to avoiding correlated errors between FFQ and others self-reported measures. In addition, the size of the sample allows investigation of the validity across important subject characteristics such as gender, age, smoking status, BMI, diploma and supplements’ use. No notable differences were observed between the different characteristics except between men and women, smokers and no smokers for vitamin E and between supplements’ users for vitamin B9 and D. Intakes of fruits and vegetables were better measured in men than in women but this did not reflect a clear gender difference in the quality of response as the correlation for micro-nutrient tend to be higher in women. Thirdly, as the recruitment of participants took place throughout the year, the four seasons were well represented and therefore the intakes of all foods were covered. Finally, the socio-demographic characteristics of participants in the validation study were very similar to those of the overall study participants (data not shown). Therefore, we can assume that the rest of the NESCAV sample performs in the same way.

The limitation of this study was that we did not use recovery biomarkers. Validation study should ideally be carried out using recovery biomarkers such as doubly labeled water, markers of potassium and nitrogen in 24 h urine collections to validate total energy intake, potassium and protein intake respectively. Recovery biomarkers are considered the gold standard but the availability and expense of those biomarkers made their use not possible for the validation of this questionnaire.

In summary, results of the biomarker-analyses, in combination with our previous findings from comparison between the FFQ and DR allow to evaluate the overall validity of our FFQ. In the previous study comparing estimates of several macro- and micro-nutrients computed from the FFQ and DR, the relationships between the two measurement tools were satisfactory [15]. However, Results for protein, cholesterol, starch, vitamins A, E and B12 ought to be interpreted with caution. In the present validation study, our FFQ performed well in ranking most of micro-nutrient, particularly fruits and vegetables intakes. Worth noted, despite the absence of agreement for vitamin B12 in the comparison with DR, significant correlations were observed in biomarkers study. However, sodium and vitamin D and E will have to be use carefully since no significant associations were observed after adjustment.

## Conclusion

The present findings, combined with those previously published against DR suggest that the FFQ permits to rank people living in the Greater region, according to nutrient intake, in both men and women, and is suitable to be used in the NESCAV study. Nonetheless results for protein, cholesterol, sodium, starch and vitamin A, E, and D should be interpreted with caution. The fact that the measures of agreement differed between nutrients and between men and women indicate that there can be no single measure of validity of a given FFQ for all subjects and all nutrients.

## Abbreviations

FFQ: Food frequency questionnaire; CVRF: Cardiovascular risk factors; LDL: Plasma low density cholesterol; HDL: Plasma high density cholesterol; BMI: Body mass index; SFA: Saturated fatty acids.

## Competing interests

The authors declare that they have no competing interests.

## Authors’ contribution

All the authors were involved in the conception and design of the validation study; NS performed the statistical data analyses and drafted the first version of the manuscript with AAlk. MG coordinated the field data collection. MG and AAlb contributed to the critical revision of the manuscript. All of the authors reviewed drafts and approved the final version of the manuscript.

## Supplementary Material

Additional file 1: Table S1 Laboratory analyses of biomarkers. Click here for file
